# The effects of spousal migration on perinatal healthcare utilization

**DOI:** 10.1186/s12884-023-05590-w

**Published:** 2023-06-12

**Authors:** Angubeen Gul Khan, Heidi West, Abdur Razzaque, Randall Kuhn

**Affiliations:** 1 Department of Community Health Sciences, University of California, Los Angeles, USA; 2 Department of Health Policy and Management, University of California, Los Angeles, USA; 3 Health System and Population Studies Division, International Centre for Diarrhoeal Disease Research, Bangladesh (Icddr,B), Dhaka, Bangladesh

**Keywords:** Migration, Pregnancy, Gender, Healthcare, Bangladesh, Maternity, LMIC

## Abstract

Over the last three decades, out-migration has become a stable source of income for more than 12 million Bangladeshis. Of those migrants, 90% are men. Due to patriarchal cultural norms in Bangladeshi society, the migration of a male spouse may have significant consequences for the social well-being and health of left-behind women. In this study, we examine the impact of external (out of country) and internal (rural to urban) spousal migration on the perinatal healthcare utilization of left-behind women. We used data from the 2012 Matlab Health and Socioeconomic Survey (MHSS2) to examine use of antenatal care, presence of a medically qualified attendant at birth, and delivery at a healthcare facility for live births that occurred between 2007 and 2014 for currently married women aged 15-45 (*N* = 1,458 births among 1,180 women). Adjusted regression models indicated that for births occurring to women with a migrant spouse, odds of receiving antenatal care were significantly higher (OR: 4.1 for births to women with a domestic urban migrant spouse and 4.6 for births to women with an international migrant spouse, *p* < 0.01). Spousal migration was not linked to having a medically qualified attendant at birth or delivery at a clinic or hospital. Results suggest that spousal migration may be beneficial for receiving health care during a pregnancy, but not for the type of attendant or place of delivery at birth.

**Supplementary Information:**

The online version contains supplementary material available at 10.1186/s12884-023-05590-w.

## Background

In pursuit of a shared goal, the Bangladeshi national government, United Nations Population Fund (UNFPA), United Nations Children’s Fund (UNICEF), and World Health Organization (WHO), collaborated to help Bangladesh achieve great success in meeting their Millennium Development Goals for maternal and neonatal mortality [1]. However, disparities in maternal and neonatal health outcomes persist in Bangladesh because of geographic and socioeconomic disparities such as rural–urban residence and wealth status [2]. The geographic and socioeconomic disparities in health care in Bangladesh may be attributed to several factors. Foremost**,** the Bangladeshi healthcare system suffers from a misallocation of resources to adequately service the population. Most physicians and healthcare workers in the country are concentrated in urban areas, while more populous rural areas often lack proper health care facilities. Though national government-funded hospitals in rural areas provide less expensive treatment to rural citizens, these hospitals are often poorly funded, understaffed, and overcrowded due to the limited number of facilities in rural areas [3]. Additionally, the health care system is decentralized and partially run by for-profit health care and pharmaceutical companies [4]. Therefore, access to health services entails high out-of-pocket payments, creating a cost barrier for health care among poor Bangladeshi citizens.

One way to reduce geographic and socioeconomic disparities in maternal and infant health care in Bangladesh is through spousal migration. Each year, over half a million individuals leave the country or leave rural villages for urban settings in search of temporary labor opportunities [5]. At the macro-level, remittances from migrants significantly improved the national economy [6]. At the micro-level, studies have documented the positive impact of migration on children’s and sibling’s schooling, on the health of older parents, and on the empowerment and healthcare utilization of women [7, 8]. Similarly, among left-behind women, migration may offer a flow of foreign income that can alleviate economic barriers to maternal health care in rural areas of Bangladesh. In this study, we examined the role of spousal migration on healthcare utilization during the perinatal period. We also examined the mediating role of remittances on the associations between spousal migration and healthcare utilization outcomes. Additionally, we analyzed other potential social and relational mediators of the relationship between spousal migration and perinatal healthcare, such as living in a multigenerational home and having regular communication with one’s partner.

In Bangladesh today, 95% of family members who live outside of the household are men [9] and 12% of married women have a migrant spouse [10]. As out-migration has increased globally, especially in the global south, it has gained recognition as a critical determinant of health [11]. Past studies find spousal migration to be associated with negative and positive health outcomes for left-behind women. For example, some studies report that left-behind women exhibit higher levels of stress, depression, and anxiety [12–17] and engage in lower levels of communication with their partners on issues of family planning and childbearing [18]. However, studies conducted in Bangladesh and Sub-Saharan Africa found that spousal migration was directly associated with increased rates of general and maternal and child healthcare utilization [19, 20]. Changes in healthcare utilization among left-behind women may relate to changes in a woman’s positionality in the household after the migration of a spouse. For example, left-behind women who receive remittances from their spouse may gain financial resources that could improve their social standing in the community and permit them to access maternal and child healthcare services [21–25]. However, this may be conditional on one’s living situation. For example, left-behind women who live in multigenerational households with their natal family or in-laws may face gendered barriers that reduce their ability to access health care [26]. While there are studies that show how decision-making power and empowerment is elevated among left-behind women relative to women who lived with their spouses [17, 27–31], there are also reports that left-behind women experience increased odds of delaying and forgoing health care during pregnancy if they also have lower decision-making power [32, 33].

Family members can also influence left-behind women’s healthcare utilization [19]. For example, compared to women who live alone, women who live in a multigenerational home (i.e., with their in-laws or natal family) may have difficulty obtaining permission to travel to health facilities alone or face pressure to have a home birth [29, 34–39]. Migrant spouses who maintain financial control in their household and family matters, or through non-monetary means, such as regular communication with their wife, may be able to mitigate pressures from natal family or in-laws to have a home birth [28].

**Fig. 1:**
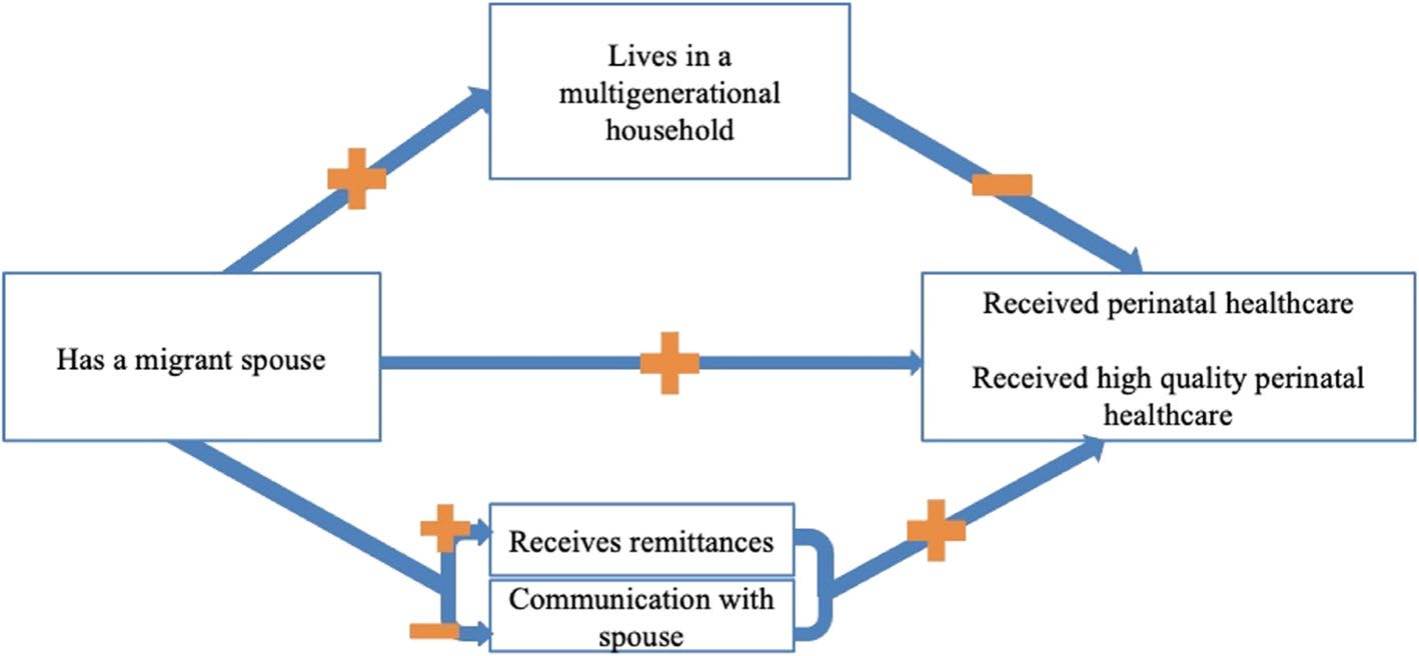
Conceptual model of spousal migration and perinatal healthcare utilization

In this study, we use Anderson’s model for health service utilization, which posits that predisposing factors, enabling resources, structural conditions, and need influence utilization of health services [40]. In this study spousal migration is considered a structural determinant of perinatal healthcare utilization, while monetary resources (e.g., remittances) and social support (e.g., regular communication with a spouse or living in a multigenerational household) are considered enabling resources. Remittances and spousal communication are expected to mediate the effects of spousal migration on perinatal healthcare utilization. Alternatively, living in a multigenerational home is expected to dampen the potential benefits of out-migration on perinatal healthcare utilization among left-behind women (Fig. 1).

## Method

### Data source

The study used data from Matlab, which is a rural district in Bangladesh about 60 kilometers south of Dhaka, with a population over 200,000 that spans 149 villages. The study area was the site for the Maternal Child Health/Family Planning (MCH/FP) Program that was initiated in Matlab in 1977 by the International Center for Diarrheal Disease Research, Bangladesh (icddr,b). This program has had notable positive effects on women's health in the area, including substantial changes in lifetime contraceptive use and fertility behaviors [41].

Villages across the study area were assigned to treatment and non-treatment group blocks based on their social and economic similarity and their distance from transportation and healthcare facilities [42, 43]. The study sample was identified using the Matlab Health and Demographic Surveillance System (MHDSS), which is a unique longitudinal demographic registration system that has recorded all births, deaths, marriages, and migrations occurring in the area since 1974. The first Matlab Health and Socioeconomic Survey sampled 7% of households included in the MHDSS in 1996 (MHSS1) [41]. This study primarily used MHSS2, which was a panel follow-up of MHSS1 conducted from 2012 to 2014. MHSS2 includes data from over 90% of households that were surveyed in MHSS1 [41]. Protection of human participants during fieldwork and data analysis was ensured under the icddr,b Ethical Review Committee Protocol #PR-10005.

### Sample

MHSS2 included 3,933 currently married women between the ages of 15 and 45 years who responded to the child survey (had at least one live or non-live birth). The sample was then restricted to women who had a live birth between 2007 and 2014 and those living in comparison areas of the existing MCH/FP program. The study includes births that occurred in non-treatment areas to mitigate program effects and self-selection bias for in-migration areas. The final sample included 1,180 women who cumulatively had 1,458 live births. Additional file 1 shows the screening process used to achieve the final analytic sample.

### Measures

The dependent variables of the analysis include three dimensions of perinatal healthcare utilization: 1) receiving antenatal care, 2) having a medically qualified attendant present at birth, and 3) delivery at a health care facility. To assess if the respondent received antenatal care for a pregnancy, respondents were asked to report if they had a pregnancy check-up for each live birth (0 = no, 1 = yes). Respondents then indicated if they had a medically qualified attendant present for each live birth by choosing if one of sixteen type of birth attendants was present during the birth. This item was recoded as a dichotomous variable (0 = not medically qualified, which included a pharmacist, community health worker, village doctor or healer, trained or traditional midwife, family member, or friend; 1 = medically qualified, which included doctor, nurse, or paramedic). Though recent studies indicate that midwives who receive skilled training are often considered to be a part of the formal health sector, the first class of midwives in Bangladesh who were trained according to international standards did not graduate before 2013 [44]. In this study, the sample is limited to births that occurred from 2007 to 2014, and most births occurred prior to implementation of international standards. Therefore, we conceptualized both trained and traditional midwives as part of the informal sector and classified them as “not medically qualified”.

Finally, respondents indicated if delivery occurred at a healthcare facility by reporting the location where each live birth occurred. Responses included a private, government-funded, or icddr,b hospital or clinic, community welfare center, pharmacy, or home. The responses were recoded into a dichotomous measure (0 = delivery did not occur at a healthcare facility, but rather their home, someone else’s home, or a pharmacy; 1 = delivery occurred at a healthcare facility, such as a hospital or clinic).

The independent variable was a three-level categorical measure of the husband’s migration status (0 = non-migrant spouse, 1 = domestic urban migrant spouse, and 2 = international migrant spouse) at the time of MHSS2. This variable was created based on items related to the spouse’s location relative to the respondent (e.g., residence in the same household, bari, thana, or village or outside of the household in a different bari, thana, village, or country). If the spouse was living outside of the household in a different village or country, we used their indicated village or country code to determine if they were a domestic urban or international migrant spouse. If the spouse was living outside of the household for reasons other than migration (approximately 2% lived outside of the household due to separation, recent marriage in which households had not yet joined, living with another wife’s family in cases of polygamy, or residence outside the household in a rural district), they were recoded into the non-migrant spouse category which was also the modal category.

Mediation analysis included measures of living in a multigenerational household, receiving remittances, and spousal communication. Living in a multigenerational household was coded as a three-level categorical variable (0 = living as the head of a household, 1 = living with natal family, or 2 = living with in-laws) and based on the household roster. Receipt of remittances was measured using a binary item in which respondents indicated whether they had received money from their spouse in the past year (0 = no, 1 = yes). Receipt of remittances was measured using a binary measure rather than a continuous measure of monetary value since values could vary throughout the year and remittances received in prior years could be used for perinatal health care (West et al., 2021). Finally, respondents reported spousal communication through a measure that assessed how often they were in contact with their spouse (by telephone, text message, email or post) in the past year (0 = less than daily, 1 = daily).

Covariates were selected based on their known linkage to migration within the household and use in studies that have used the same data [19]. For example, out-migration to internal and international destinations in MHSS2 was higher among younger male cohorts. As a result, our multivariate models controlled for respondent age. Prior studies also indicate that migrant household members typically have more years of education and greater land assets relative to members of non-migrant households [45]. Therefore, our models also control for factors such as respondents’ and spouses’ years of schooling (a four-level categorical measure of school attendance that ranged from 0 to 10 or more years) and household assets (log transformation of prior household assets based on the sum value of assets across all productive and non-productive types). We also controlled for distance of residence to the nearest healthcare facility as well as generational controls from the original MHSS1 including one’s own migration history and family’s history of migration (father and brother) to further strengthen the analysis. Missing responses (under 10%) were recoded into the modal category.

### Statistical analysis

Analyses were conducted using Stata 15 and included univariate, bivariate, and multivariate regression analyses. Univariate analysis and bivariate analysis of sample characteristics were conducted among women at the individual-level. Bivariate and multivariate analysis of the perinatal healthcare utilization outcomes were conducted at the birth-level. Univariate statistics and bivariate associations were examined using Pearson’s chi-square test and independent t-tests to estimate if there were significant differences in sample characteristics and perinatal healthcare utilization outcomes by spousal migration status. Then, we conducted unadjusted and adjusted binomial logistic regressions to examine if spousal migration was associated with receiving antenatal care, having a medically qualified assistant at birth, and delivery at a healthcare facility. Finally, we estimated multivariate regression models and predicted probabilities to assess interaction effects of living in a multigenerational household on the associations between the perinatal healthcare utilization outcomes by spousal migration status. All multivariate regression analyses were weighted for representativeness of the 2012 population of the Matlab HDSS area. Using the full HDSS in 2012, representative weights were constructed based on Monte Carlo simulations which estimated the probability that an individual could have been picked based on the MHSS panel sampling scheme.

## Results

Between 2007 and 2014, 1,180 currently-married women within non-treatment areas of the MHSS2 study region had 1,458 live births (Table 1). Over half of the respondents (56%) were between the ages of 25–34 and 14% had at least 10 years of schooling. On average, the respondents in this sample lived 5 kilometers from the nearest hospital, but less than 0.25 kilometers from a clinic. Almost 70% of the respondents had never lived outside of Matlab and 20% had prior family history of migration. Over 30% of respondents in the sample had a migrant spouse; 11% (*n* = 133) had a domestic urban migrant spouse and 20% (*n* = 236) had an international migrant spouse. Nearly half of the sample lived with their natal family or in-laws, but among left-behind women, living with one’s natal family or in-laws was significantly more common. Approximately 90% of left-behind women received some form of remittances and between 61% to 71% communicated with their spouses every day (71% if spouse was a domestic urban migrant and 61% if spouse was an international migrant).

**Table 1:** Sample characteristics of currently-married women (15–45 years) who had a live birth between 2007–2014 in non-treatment areas of Matlab, Bangladesh (*N* = 1,180 women)

	Total		Non-migrant spouse		Domestic urban migrant spouse		International migrant spouse		*p*-value
	*N* = 1,180		*N* = 811 (68.7%)		*N* = 133 (11.3%)		*N* = 236 (20.0%)		
	N	(%)	N	(%)	N	(%)	N	(%)	
Age									
15–24	347	(29.4%)	213	(26.3%)	51	(38.3%)	83	(35.2%)	*
25–34	655	(55.5%)	473	(58.3%)	63	(47.4%)	119	(50.4%)	
35–45	178	(15.1%)	125	(15.4%)	19	(14.3%)	34	(14.4%)	
Years attended school									
0 years	85	( 7.2%)	76	( 9.4%)	5	( 3.8%)	4	( 1.7%)	***
1–4 years	217	(18.4%)	171	(21.1%)	18	(13.5%)	28	(11.9%)	
5–9 years	709	(60.1%)	459	(56.6%)	89	(66.9%)	161	(68.2%)	
10 + years	169	(14.3%)	105	(12.9%)	21	(15.8%)	43	(18.2%)	
Years that spouse attended school									
0 years	209	(17.7%)	177	(21.8%)	12	( 9.0%)	20	( 8.5%)	***
1–4 years	258	(21.9%)	197	(24.3%)	24	(18.0%)	37	(15.7%)	
5–9 years	512	(43.4%)	315	(38.8%)	62	(46.6%)	135	(57.2%)	
10 + years	201	(17.0%)	122	(15.0%)	35	(26.3%)	44	(18.6%)	
Log transformation of total household assets ($)	13.2	(1.8)	12.9	(1.9)	13.4	(1.4)	14.0	(1.4)	***
Distance to the nearest health facility (km)									
Hospital	5.4	(4.3)	5.4	(4.4)	6.0	(4.0)	5.0	(3.8)	
Clinic	0.2	(0.3)	0.2	(0.3)	0.2	(0.3)	0.2	(0.3)	*
Pharmacy	0.6	(0.7)	0.6	(0.7)	0.6	(0.8)	0.6	(0.7)	0.47
Ever lived outside of Matlab	377	(31.9%)	266	(32.8%)	50	(37.6%)	61	(25.8%)	*
Father was an international migrant^1^	66	( 5.6%)	40	( 4.9%)	9	( 6.8%)	17	( 7.2%)	0.34
Sibling was an international migrant^1^	249	(21.1%)	154	(19.0%)	28	(21.1%)	67	(28.4%)	**
Lives in a multigenerational household									
Head of household/wife	629	(53.3%)	511	(63.0%)	41	(30.8%)	77	(32.6%)	***
Lives with natal family	96	( 8.1%)	25	( 3.1%)	32	(24.1%)	39	(16.5%)	
Lives with in-laws	455	(38.6%)	275	(33.9%)	60	(45.1%)	120	(50.8%)	
Received remittances	351	(29.7%)	20	( 2.5%)	118	(88.7%)	213	(90.3%)	***
Daily spousal communication	249	(21.1%)	10	( 1.2%)	95	(71.4%)	144	(61.0%)	***

Notes: 1) Generational controls of family migration were derived from the Matlab Health and Socioeconomic Survey conducted in 1996 (MHSS1). 2) Pearson chi-square tests were used to estimate bivariate difference in age, years attended school, years that spouse attended school, distance to the nearest healthcare facility, history of migration (for self, father, and sibling), living in a multigenerational household, receiving remittances, and daily spousal communication by spousal migration status. Independent t-test was used to assess bivariate differences in the log transformation of total household assets by spousal migration status. 3) *p < 0.05; **p < 0.01; ***p < 0.001

Receiving antenatal care was high in this sample (69%) but was significantly greater for pregnancies which occurred among left-behind women (78% if spouse was domestic urban migrant and 82% if spouse was an international migrant; *p* < 0.001) (Table 2). Having a medically qualified attendant present at birth was significantly more common for births among women with an international migrant spouse (33%) relative to those with a domestic urban migrant spouse (26%) or non-migrant spouse (24%) (*p* < 0.01). The bivariate results revealed similar trends for the place of delivery. Only 21% of births among women with a non-migrant spouse were delivered at a healthcare facility, compared to 34% of births to women with an international migrant spouse (*p* < 0.001).

**Table 2:** Perinatal healthcare utilization outcomes of currently-married women ages 15–45 years who had a live birth between 2007–2014 in non-treatment areas of Matlab, Bangladesh by spousal migration status (*N* = 1,458 births)

	Total		Non-migrant spouse		Domestic urban migrant spouse		International migrant spouse		*p*-value
	*N* = 1,458		*N* = 1,013		*N* = 160		*N* = 285		
	N	(%)	N	(%)	N	(%)	N	(%)	
Received antenatal care	1,006	(69.0%)	648	(64.0%)	124	(77.5%)	234	(82.1%)	***
Type of birth attendant at birth^1^									
Not a medically qualified attendant	1,081	(74.1%)	773	(76.3%)	118	(73.8%)	190	(66.7%)	**
Medically qualified attendant	377	(25.9%)	240	(23.7%)	42	(26.3%)	95	(33.3%)	
Delivery at a healthcare facility^2^									
No	1,110	(76.1%)	804	(79.4%)	118	(73.8%)	188	(66.0%)	***
Yes	348	(23.9%)	209	(20.6%)	42	(26.3%)	97	(34.0%)	

Notes: 1) Not medically qualified attendants include community health workers, trained and traditional midwives, traditional healers, village doctors, pharmacist, family members and friends or other unspecified and medically qualified include nurses, paramedics, and doctors with an MBBS degree; 2) No includes own home or someone else's home, pharmacy, or other unspecified locations; 3) Pearson chi-square tests were used to estimate bivariate differences in perinatal healthcare utilization outcomes by spousal migration status. 4) *p < 0.05; **p < 0.01; ***p < 0.001

### Received antenatal care

**Fig. 2:**
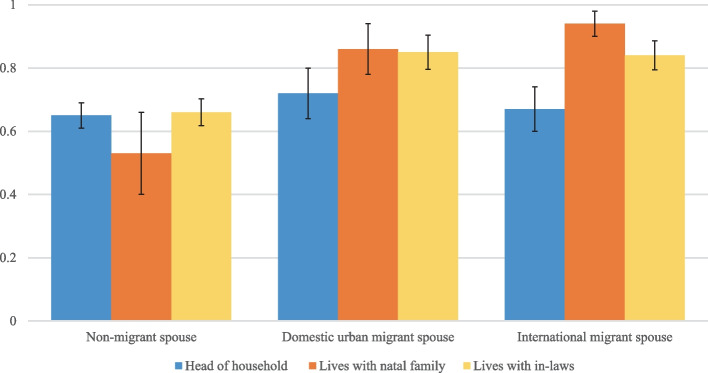
Predicted probabilities of receiving antenatal care by spousal migration status and living in a multigenerational household (*N* = 1,458 births)

For births among left-behind women with domestic urban (OR: 3.0; *p* < 0.001) or international migrant spouses (OR: 2.2; *p* < 0.01), receiving antenatal care was significantly higher than for births to women with non-migrant spouses (Table 3, Models 1 and 2). Upon adjusting for covariates, left-behind women with domestic urban or international migrant spouses had over four times the odds of receiving antenatal care compared to women with a non-migrant spouse (Table 3, Model 3; *p* < 0.01). Additionally, relational factors significantly modified these associations. For example, living in a multigenerational household significantly mediated the association between having a migrant spouse and receiving antenatal care. That is, among left-behind women whose spouses were international migrants, living with one’s natal family or in-laws increased the probability of receiving antenatal care compared to births among those who lived alone (see Fig. 2). Interaction models did not indicate significant mediation effects of remittances on the association between having a migrant spouse and receiving antenatal care. Finally, the odds of receiving antenatal care increased for births occurring to left-behind women who also communicated with their spouse daily (*p* < 0.001).

**Table 3:** Weighted binomial logistic regression models of received antenatal care by type of migrant spouse for births occurring to currently-married women (15–45 years) who had a live birth between 2007–2014 in non-treatment areas of Matlab, Bangladesh (*N* = 1,458)

	Model 1	Model 2	Model 3	Model 4	Model 5	Model 6
	*N* = 1,458	*N* = 1,458	*N* = 1,458	*N* = 1,458	*N* = 1,458	*N* = 1,458
	OR (s.e.)	OR (s.e.)	AOR (s.e.)	AOR (s.e.)	AOR (s.e.)	AOR (s.e.)
Migrant spouse						
Domestic urban migrant	3.000*** (0.806)	3.339* (1.662)	4.115** (2.086)	1.995 (1.267)		
International migrant	2.178** (0.560)	3.204* (1.495)	4.606** (2.258)	1.807 (1.074)		
Household structure						
Lives with natal family		2.467* (0.897)	1.931 (0.914)	0.676 (0.537)	1.583 (0.723)	1.927 (0.908)
Lives with in-laws		2.066*** (0.395)	1.420 (0.299)	1.069 (0.233)	**1.417 + **(0.299)	**1.416 + **(0.298)
Received remittances		0.342* (0.160)	0.322* (0.158)	0.456 (0.241)		0.354* (0.165)
Daily spousal communication		2.188* (0.804)	1.911 (0.769)	1.926 (0.752)	1.715 (0.685)	
Migrant spouse x household structure						
Domestic urban migrant x lives with natal family				4.008 (4.717)		
Domestic urban migrant x lives with in-laws				2.320 (1.540)		
International migrant x lives with natal family				14.69* (18.93)		
International migrant x lives with in-laws				2.962 (1.724)		
Migrant spouse x remittances (ref. non-migrant spouse)						
Domestic urban migrant x did not receive remittances					2.829 (2.069)	
Domestic urban migrant x received remittances					1.610 (0.664)	
International migrant x did not receive remittances					1.902 (1.200)	
International migrant x received remittances					1.716 (0.584)	
Migrant spouse x spousal communication (ref. non-migrant spouse)						
Domestic urban migrant x less than daily spousal communication						**2.798 + **(1.529)
Domestic urban migrant x daily spousal communication						8.301*** (4.685)
International migrant x less than daily spousal communication						4.283** (2.192)
International migrant x daily spousal communication						7.785*** (4.589)
Constant	1.795*** (0.166)	1.410** (0.156)	0.183* (0.131)	0.174* (0.126)	0.188* (0.135)	0.180* (0.129)
Wald chi2	23.160	53.930	174.020	195.900	173.870	175.310
Pseudo R2	0.028	0.058	0.224	0.232	0.219	0.225

Notes: 1) Models 3–6 control for age, years attended school, spouse’s years attended school, log transformation of productive and nonproductive assets, self and family history of migration (father and sibling), distance from a healthcare facility, and year of birth. 2) *** *p* < 0.001, ** *p* < 0.01, * *p* < 0.05

### Medically qualified attendant present at birth

Left-behind women with a domestic urban migrant spouse had nearly twice the odds of having a medically qualified attendant present at birth (Table 4, Model 1; *p* < 0.05). However, after adjusting for living in a multigenerational household, receiving remittances, and daily communication with one’s spouse, the association was no longer significant (Table 4; Model 2). Additionally, although unadjusted regression models (Table 4, Model 2) indicated that there were significant effects of living with one’s in laws (OR: 1.5; *p* < 0.05) and daily communication with a spouse (OR: 2.0; *p* < 0.5) on having a medically qualified attendant present at birth (*p* < 0.05), the effects were not significant after adjusting for covariates (Table 4, Model 3). Living in a multigenerational household did not have significant mediating effects on the association between spousal migration and having a medically qualified attendant present at birth (Table 4, Model 4).

**Table 4:** Weighted binomial logistic regression models of having a medically qualified attendant present at birth by type of migrant spouse for births occurring to currently-married women (15–45 years) who had a live birth between 2007–2014 in non-treatment areas of Matlab, Bangladesh (*N* = 1,458 births)

	Model 1	Model 2	Model 3	Model 4
	*N* = 1,458	*N* = 1,458	*N* = 1,458	*N* = 1,458
	OR (s.e.)	OR (s.e.)	AOR (s.e.)	AOR (s.e.)
Migrant spouse				
Domestic urban migrant	1.905* (0.592)	1.169 (0.759)	1.200 (0.669)	0.544 (0.455)
International migrant	1.218 (0.294)	0.912 (0.468)	0.946 (0.440)	1.272 (0.750)
Household structure				
Lives with natal family		1.953 (0.674)	1.144 (0.438)	1.611 (1.195)
Lives with in-laws		1.516* (0.292)	0.903 (0.193)	0.887 (0.219)
Received remittances		0.735 (0.358)	0.926 (0.423)	0.886 (0.411)
Daily spousal communication		2.032* (0.733)	1.476 (0.528)	1.545 (0.542)
Migrant spouse x household structure				
Domestic urban migrant x lives with natal family				1.271 (1.418)
Domestic urban migrant x lives with in-laws				3.339 (2.849)
International migrant x lives with natal family				0.590 (0.573)
International migrant x lives with in-laws				0.660 (0.356)
Constant	0.318*** (0.0341)	0.267*** (0.0391)	0.0204*** (0.0189)	0.0246*** (0.0224)
Wald chi2	4.550	15.250	120.430	128.400
Pseudo R2	0.008	0.024	0.164	0.169

Notes: 1) Models 3 and 4 control for age, years attended school, spouse’s years attended school, log transformation of productive and non-productive assets, self and family history of migration (father and sibling), distance from a healthcare facility, and year of birth. 2) *** *p* < 0.001, ** *p* < 0.01, * *p* < 0.05

### Delivery at a healthcare facility

Bivariate analyses indicated that having a domestic urban migrant spouse was significantly linked to place of delivery (Table 5, Model 1), however, the association was not significant in adjusted models. Unadjusted models also indicated that there were significantly greater odds of delivering at a healthcare facility for births to respondents who lived with their natal family (OR: 2.1, *p* < 0.05) or communicated with their spouse daily (OR: 2.3, *p* < 0.05; Table 5, Model 2). However, these links are not significant after controlling for other covariates. Additionally, we found no mediation effects of living in a multigenerational household on the association between spousal migration status and delivery at a healthcare facility (Table 5, Model 4).

**Table 5:** Weighted logistic regression models of delivering at a clinic or hospital by type of migrant spouse for births occurring to currently-married women (15–45 years) who had a live birth between 2007–2014 in non-treatment areas of Matlab, Bangladesh (*N* = 1,458 births)

	Model 1	Model 2	Model 3	Model 4
	*N* = 1,458	*N* = 1,458	*N* = 1,458	*N* = 1,458
	OR (s.e.)	OR (s.e.)	AOR (s.e.)	AOR (s.e.)
Migrant spouse				
Domestic urban migrant	1.928* (0.612)	0.548 (0.232)	0.561 (0.242)	0.273 (0.226)
International migrant	1.507 (0.369)	0.537 (0.210)	0.552 (0.223)	0.647 (0.383)
Household structure				
Lives with natal family		2.140* (0.757)	1.425 (0.556)	1.481 (1.126)
Lives with in-laws		1.454 (0.294)	0.941 (0.211)	0.917 (0.238)
Received remittances		1.521 (0.552)	2.011 (0.769)	2.027 (0.884)
Daily spousal communication		2.336* (0.836)	1.666 (0.605)	1.702 (0.613)
Migrant spouse x household structure				
Domestic urban migrant x lives with natal family				1.750 (1.985)
Domestic urban migrant x lives with in-laws				2.751 (2.408)
International migrant x lives with natal family				0.875 (0.851)
International migrant x lives with in-laws				0.762 (0.424)
Constant	0.249*** (0.0284)	0.207*** (0.0326)	0.0134*** (0.0130)	0.0151*** (0.0146)
Wald chi2	6.090	23.110	114.400	120.800
Pseudo R2	0.011	0.036	0.160	0.163

Notes: 1) Models 3 and 4 control for age, years attended school, spouse’s years attended school, log transformation of productive and non-productive assets, self and family history of migration (father and sibling), distance from a healthcare facility, and year of birth. 2) *** *p* < 0.001, ** *p* < 0.01, * *p* < 0.05

## Discussion

This study indicates that left-behind women have greater odds of receiving antenatal care during pregnancy compared to women who did not have a migrant spouse and that the benefits of spousal migration are not only driven by remittances, but may be socially motivated. For example, this study indicates that more so than remittances, left-behind women who live in a multigenerational household and have regular contact with their spouse have greater odds of receiving antenatal care. The positive effects of spousal migration did not extend to having a medically qualified attendant present at birth or delivery at a healthcare facility. In fact, most women (over 65%), regardless of their husband’s migration status, delivered with someone who was not medically qualified — most commonly a traditional midwife — and at home.

Prior studies of spousal migration focus on the role of remittances as the primary driver of migration-related health and social benefits that are experienced by family members who are left behind. However, this study found that the benefits of migration for perinatal healthcare utilization are associated with social factors related to the left-behind women’s living situation, such as living with one’s natal family or regularly communicating with their spouse. Our findings contrast with prior studies that have found that remittances in the form of money transfers can help improve the lives of left-behind women [46, 47]. However, some studies have also shown that left-behind women receive minimal and sporadic money transfers from their migrant partners, highlighting how left-behind women are often marooned in financially precarious positions and with limited freedom and mobility [48]. Additionally, left-behind women who live in multigenerational households may be expected to share remittances sent to them with other members of the household, leaving little to directly benefit them [49]. Finally, our analyses may not have picked up the potential benefits of remittance on left-behind women’s perinatal healthcare utilization due to temporal differences in the measurement of remittances (past 12 months) relative to when antenatal care was received (any time between 2007 to 2014).

Our findings indicate that family structure was one of the drivers of perinatal healthcare utilization for left-behind women. This conflicts with past studies reporting that the absence of a migrant spouse can lead to a fragmented family structure [14, 50–53], and that many of the women are left to live with their in-laws and exposed to excessive surveillance and patriarchal values and expectations [52, 54, 55]. The benefits of living in a multigenerational household were observed among women who were living with their natal family. In contrast with left-behind women who lived with their in-laws, left-behind women who lived with their natal family may have had additional social support and someone to accompany them to their antenatal care visits. Future studies should examine if there are differences within the multigenerational household context for left-behind women who are living with their in-laws versus their natal family.

Daily communication with a partner also amplified the positive effects of spousal migration on receiving antenatal care. Past studies have found that migrant men who were in consistent communication with their left-behind wives were able to keep strong ties with their family [18]. In the context of perinatal health care, regular communication may allow a migrant spouse to be involved in his wife’s pregnancy and help a left-behind woman advocate for her perinatal healthcare needs [28].

The prevalence of home births and delivery with a midwife in this population suggests the absence of a perceived need for medically trained professionals or delivery at a healthcare facility [56]. The need for a skilled attendant at birth is not strongly recognized in many rural areas of Bangladesh [57]. In fact, the difference between traditional midwives and skilled midwives is unclear to expectant mothers. There is instead a preference for a birth attendant who is familiar with family customs and beliefs and exhibits strong interpersonal communication [58]. As consequence, general birth planning and preparedness in rural areas of Bangladesh remains low. This may explain why although the migration of a spouse was observed to have positive effects on utilization of antenatal care services, similar effects were not observed for having a medically qualified attendant at birth or delivery at a healthcare facility. Although Matlab is the site of a larger maternal and child health intervention and may be unique from other rural areas in the country, these attitudes may still persist because of rigid and traditional social roles that are prevasive in the country.

This study has some limitations. We were unable to make any causal links between migration and perinatal healthcare utilization, as our study used cross-sectional data. There were also temporal concerns regarding if a spouse was present at the time of the birth. The measure for antenatal care was also limited because we did not know if an adequate number of antenatal care visits were received throughout the pregnancy. Finally, this study does not address long-term consequences of migration and perinatal healthcare utilization for infant health. However, this could be a future area of study as the research on migration and maternal and child health care continues to grow.

Despite these limitations, the study has major strengths. Matlab was a unique and suitable setting for this study due to its high levels of out-migration. The sample was obtained through a randomized cluster sample in a high-out migration setting, which improved the external validity of the findings and made the sample a reliable representation of the Matlab area and other high out-migration affected populations. MHSS2 was conducted among all members of the household and responses can be cross validated with other respondents within a household. Further, the availability of two rounds of survey data allowed us to control for sociodemographic characteristics and migration history, which could confound the association between spousal migration and perinatal healthcare utilization outcomes. Additionally, sensitivity analyses indicated that including domestic urban migrants in the analysis was the best way to assess left-behind women’s perinatal healthcare utilization as opposed to exclusively focusing on international migration.

This study is one of few that explores healthcare utilization of left-behind women and the only known study to assess perinatal healthcare utilization among left-behind women in Bangladesh. The results indicate the ways that out-migration can help improve maternal and child health care in low-income and rural settings. Additionally, this study highlights that the benefits of out-migration are not only limited to monetary gains, but that changes in family dynamics and maintaining regular communication with a spouse can improve maternal health care access in rural areas. Interventions that aim to reduce the gap between rural and urban disparities in maternal and child health in Bangladesh should take into consideration how spousal migration effects women’s roles and dynamics in the home, relationship dynamics between partners, and how these factors contribute to healthcare utilization during pregnancy and childbirth.

## Conclusion

This study examined the role of spousal migration on perinatal healthcare utilization and found that spousal migration had positive effects on receiving antenatal care. Furthermore, this study indicated that remittances were not the sole drivers of migration benefits, but that household and relational factors like living in a multigenerational household and regular communication with one’s partner could amplify the benefits of having a migrant spouse. This study challenges the degree to which remittances help left-behind women and the fact that family and relational dynamics should be an important focus of maternal and child health care interventions for women residing in rural areas of Bangladesh. When investigating the context of a wife’s living situation and level of communication with their partner, we find that these factors can help reduce the rural–urban disparities in maternal and infant health outcomes that persist in Bangladesh.

## Supplementary Information

**Additional file 1.** Screening process for obtaining the final analytic sample of live births occurring in MHSS2 from 2007-2014.

## Data Availability

The current study utilizes a dataset made available through the California Center for Population Research at the University of California. The dataset has not been made publicly available but can be made available upon reasonable request and with permission of Dr. Randall Kuhn. These data will all be entering the public domain in the coming months, upon which we will gladly provide all code for this paper alongside that public release.

## References

[1] Koblinsky M, Anwar I, Mridha MK, Chowdhury ME, Botlero R (2008). Reducing maternal mortality and improving maternal health: Bangladesh and MDG 5. J Health Popul Nutr.

[2] Haider MR, Rahman MM, Moinuddin M, Rahman AE, Ahmed S, Khan MM (2017). Impact of maternal and neonatal health initiatives on inequity in maternal health care utilization in Bangladesh. PLoS ONE.

[3] Joarder T, Chaudhury TZ, Mannan I. Universal health coverage in Bangladesh: activities, challenges, and suggestions. La Torre G, editor. Adv Public Health. 2019;(2019):4954095. 10.1155/2019/4954095 PMC7691757 33281233

[4] Molla AA, Chi C (2017). Who pays for healthcare in Bangladesh? An analysis of progressivity in health systems financing. Int J Equity Health.

[5] Japan Social Development Fund. Bangladesh Safe Migration for Bangladeshi Workers. World Bank Accessed March 17, 2023. https://thedocs.worldbank.org/en/doc/9d8c9473e92defce23438bcf3d6a45d6-0060052022/original/BangladeshBrief-Safe-Migration-for-Bangladeshi-Workers.pdf.

[6] Etzold B, Mallick B. Bangladesh. Country Profile. 2015. https://www.researchgate.net/publication/285371220_Bangladesh_Country_Profile.

[7] Kuhn RS. A longitudinal analysis of health and mortality in a migrant-sending region of Bangladesh. In: Migration and Health in Asia. Routledge; 2006:177–208.

[8] Kuhn R (2006). The effects of fathers’ and siblings’ migration on children’s pace of schooling in rural Bangladesh. Asian Popul Stud.

[9] Bangladesh Bureau of Statistics. Population and Housing Census Preliminary Repor*t*.; 2011:19, 332. Accessed March 17, 2023. http://bbs.portal.gov.bd/sites/default/files/files/bbs.portal.gov.bd/page/7b7b171a_731a_4854_8e0a_f8f7dede4a4a/PHC2011PreliminaryReport.pdf.

[10] National Institute of Population Research and Training - NIPORT/Bangladesh, Mitra and Associates, and ICF International. Bangladesh Demographic and Health Survey 2014. Dhaka: NIPORT, Mitra and Associates, and ICF International; 2016. https://dhsprogram.com/publications/publication-fr311-dhs-final-reports.cfm.

[11] Davies AA, Basten A, Frattini C (2009). Migration: a social determinant of the health of migrants. Eurohealth.

[12] Edelblute HB, Clark S, Mann L, McKenney KM, Bischof JJ, Kistler C (2014). Promotoras across the border: A pilot study addressing depression in Mexican women impacted by migration. J Immigr Minor Health.

[13] Jin Y, Qin Q, Zhan S, Yu X, Liang L, Huang F (2016). Depressive symptoms were prevalent among left-behind women in Ma’anshan, China. J Nerv Ment Dis.

[14] Siriwardhana C, Wickramage K, Siribaddana S (2015). Common mental disorders among adult members of ‘left-behind’international migrant worker families in Sri Lanka. BMC Public Health.

[15] Sultana A (2014). Visiting husbands: Issues and challenges of women left behind. Pak J Womens Stud.= Alam-e-Niswan= Alam-i Nisvan.

[16] Yi J, Zhong B, Yao S (2014). Health-related quality of life and influencing factors among rural left-behind wives in Liuyang. China BMC Womens Health.

[17] Singh R (2018). Impact of male out-migration on women left behind: A study of two villages in Uttar Pradesh. Remittances Rev.

[18] Hendrickson ZM, Lohani S, Thapaliya Shrestha B, Underwood CR (2018). Talking about reproduction with a migrating spouse: Women’s experiences in Dhading. Nepal Health Care Women Int.

[19] West HS, Robbins ME, Moucheraud C, Razzaque A, Kuhn R (2021). Effects of spousal migration on access to healthcare for women left behind: A cross-sectional follow-up study. PLoS ONE.

[20] Atake EH (2018). The impacts of migration on maternal and child health services utilisation in Sub-Saharan Africa: evidence from Togo. Public Health.

[21] Amuedo-Dorantes C, Pozo S (2006). Migration, remittances, and male and female employment patterns. Am Econ Rev.

[22] Khan MI, Valatheeswaran C (2016). International migration, remittances and labour force participation of left-behind family members: A study of Kerala. Margin..

[23] Lokshin M, Glinskaya E (2009). The effect of male migration on employment patterns of women in Nepal. World Bank Econ Rev.

[24] Ali I, Jaleel CA, Maheshwari N, Rahman H (2019). Migration and Maternal Health Care Services Utilisation in Uttar Pradesh. India Soc Sci Spectrum.

[25] Frank R (2005). International migration and infant health in Mexico. J Immigr Health.

[26] West H. Migration, gender, and families: The effects of spousal migration on women’s empowerment (Presentation). Population Association of America Annual Research Meeting. Atlanta, GA. Presented at: Population Association of America; 2022; Atlanta, GA.

[27] Agadjanian V, Hayford SR (2018). Men’s migration, women’s autonomy, and union dissolution in rural Mozambique. J Fam Issues.

[28] Brink JH (1991). The effect of emigration of husbands of husbands on the status of their wives: An Egyptian case. Int J Middle East Stud.

[29] Desai S, Banerji M (2008). Negotiated identities: male migration and left-behind wives in India. J Popul Res.

[30] Sarker BK, Rahman M, Rahman T, Hossain J, Reichenbach L, Mitra DK (2016). Reasons for preference of home delivery with traditional birth attendants (TBAs) in rural Bangladesh: a qualitative exploration. PLoS ONE.

[31] Yabiku ST, Agadjanian V, Sevoyan A (2010). Husbands’ labour migration and wives’ autonomy, Mozambique 2000–2006. Popul Stud.

[32] Kumar A (2014). Does Male Out-Migration and Household Structure Matter in Maternal Health Services Utilization in India?. Int J Sci Res.

[33] Nabieva J, Souares A (2019). Factors influencing decision to seek health care: a qualitative study among labour-migrants’ wives in northern Tajikistan. BMC Pregnancy Childbirth.

[34] Bender DE, McCann MF (2000). The influence of maternal intergenerational education on health behaviors of women in peri-urban Bolivia. Soc Sci Med.

[35] Govindasamy P, Malhotra A (1996). Women’s Position and Family Planning in Egypt. Stud Fam Plann.

[36] Schuler SR, Rottach E (2010). Women’s Empowerment across Generations in Bangladesh. J Dev Stud.

[37] Gupta MD (1995). Life course perspectives on women’s autonomy and health outcomes. Am Anthropol.

[38] Hannaford DR. Marriage Without Borders: Transnational Spouses in Neoliberal Senegal. University of Pennsylvania/University of Pennsylvania Press; 2017.

[39] Samari G (2021). Coming back and moving backwards: return migration and gender norms in Egypt. J Ethn Migr Stud.

[40] Aday LA, Andersen R (1974). A framework for the study of access to medical care. Health Serv Res.

[41] Barham T, Champion B, Foster AD (2021). Thirty-five years later: Long-term effects of the Matlab maternal and child health/family planning program on older women’s well-being. Proc Natl Acad Sci.

[42] Fauveau V, editor. Matlab, Women, Children, and Health. Bangladesh: International Centre for Diarrhoeal Disease Research; 1994.

[43] Stinson WS, Phillips JF, Rahman M, Chakraborty J (1982). The demographic impact of the contraceptive distribution project in Matlab, Bangladesh. Stud Fam Plann.

[44] Khatun M, Akter P, Yunus S (2022). Challenges to implement evidence-based midwifery care in Bangladesh. An interview study with medical doctors mentoring health care providers. Sex Reprod Healthcare.

[45] Kikkawa A, Otsuka K (2020). The changing landscape of international migration: evidence from rural households in Bangladesh, 2000–2014. Oxf Dev Stud.

[46] Gartaula HN, Visser L, Niehof A (2012). Socio-cultural dispositions and wellbeing of the women left behind: A case of migrant households in Nepal. Soc Indic Res.

[47] McKenzie S, Menjívar C (2011). The meanings of migration, remittances and gifts: Views of Honduran women who stay. Global Netw.

[48] McEvoy J, Petrzelka P, Radel C, Schmook B (2012). Gendered Mobility and Morality in a South-Eastern Mexican Community: Impacts of Male Labour Migration on the Women Left Behind. Mobilities.

[49] Green SH, Wang C, Ballakrishnen SS, Brueckner H, Bearman P. Patterned remittances enhance women's health-related autonomy. SSM-Popul Health. 2019;9:100370. 10.1016/j.ssmph.2019.100370 PMC6978472 31993477

[50] Fernandez-Sanchez H, Salma J, Marquez-Vargas PM, Salami B (2020). Left-behind women in the context of international migration: A scoping review. J Transcult Nurs.

[51] Ikuomola AD (2015). An exploration of life experiences of left behind wives in Edo State, Nigeria. J Comp Res Anthropol Sociol.

[52] Lenoël A (2017). The, “three ages” of left-behind Moroccan wives: Status, decision-making power, and access to resources. Popul Space Place.

[53] McGuire S (2007). Martin K.

[54] Caballero M, Leyva-Flores R, Ochoa-Marín SC, Zarco Á, Guerrero C (2008). Las mujeres que se quedan: migración e implicación en los procesos de búsqueda de atención de servicios de salud. Salud pública de México.

[55] Sekhar TV (1996). Male emigration and changes in the family: impact on female sex roles. Indian J Soc Work.

[56] Perkins JE, Rahman AE, Siddique AB, Haider MR, Banik G, Tahsina T (2019). Opting for home birth in rural Bangladesh: an assessment of the current status and reasons. Birth.

[57] Tasnim S, Rahman A, Shahabuddin AK (2010). Access to skilled care at home during pregnancy and childbirth: Dhaka Bangladesh. Int Q Community Health Educ.

[58] Hlady WG, Fauveau VA, Khan SA, Chakraborty J, Yunus M (1992). Utilization of medically-trained birth attendants in rural Bangladesh. Asia Pac J Public Health.

