# Supplementary material for: The effects of spousal migration on perinatal healthcare utilization
Source: BMC Pregnancy Childbirth. 2023 Jun 12;23:434. doi: 10.1186/s12884-023-05590-w (PMC10258923; doi:10.1186/s12884-023-05590-w)

Additional file 1. Screening process for obtaining the final analytic sample of live births occurring in MHSS2 from 2007-2014


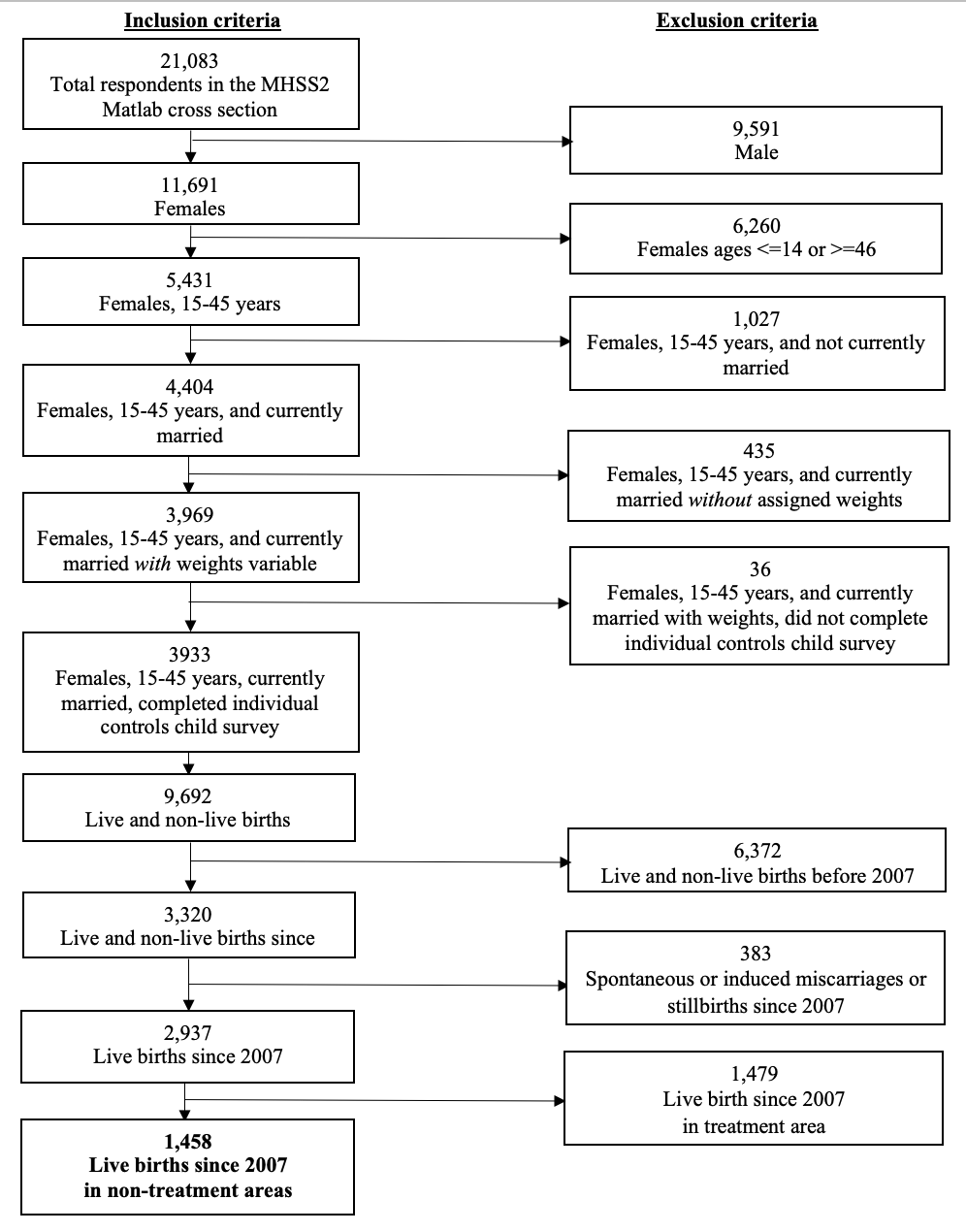

Supplement: Supplementary file 1 — Additional file 1. Screening process for obtaining the final analytic sample of live births occurring in MHSS2 from 2007-2014. [file 12884_2023_5590_MOESM1_ESM.docx]
